# Given Dimensions of Neoplastic Events as Aberrantly Operative Alternative Splicing

**DOI:** 10.4061/2010/509245

**Published:** 2009-10-12

**Authors:** Lawrence M. Agius

**Affiliations:** ^1^27 “Ballarat”, Guzeppe Caruana Str., Tal-Virtu, Rabat RBT09, Malta; ^2^Department of Pathology, Mater Dei Hospital, Tal-Qroqq, University of Malta Medical School, Msida MSD 2090, Malta

## Abstract

The provision of dynamic splicing events constitutes the reflected nature of neoplasia that locally infiltrates and systemically spreads in terms of evolutionary attributes of the primary and various secondary pathways in malignant transformation. The significant diversity in molecular characterization of the given tumor lesion would adaptively conform to dynamics of splicing as enhanced or silenced exons of the premessenger RNA molecule. The proteins synthesized are in turn potential modifiers in further gene expression within such contexts as RNA:protein and RNA:DNA binding events. The recognition of pathways of incremental scope would underline the development of lesions, such as tumors, as multiple alternative splicing phenomena primarily affecting molecular physicochemical identity. It is within contexts of operative intervention and modification that the real identity of the malignant neoplastic process arises, within terms of reference of contextual splicing events. Disrupted gene expression is thus a referential pathway in the modification of splicing that may prove constitutive or alternative, in first instance, but also aberrant as the lesion progresses locally and systemically.

## 1. Introduction

Gene expression profiles and their regulation illustrates additional layers in regulatory control of protein synthesis that implicate especially the relatively important roles of constitutive and alternative splicing of exons of premessenger RNA.

The spliceosome machinery, in particular, would constitute an expressive mechanism in the further evolution of splicing enhancement or silencing [1]. It is the absence of mutations in many disease entities, such as those characterized by tau neurofibrillary tangle formation, that would indicate a central role, in disease, for alternative splicing and for the aberrant role of alternative exons in pre-mRNA synthesis.

The overall dimensions of regulation of selection would concern in particular the exclusion of various exons and noncoding introns in the subsequent generation of messenger RNA transcripts and novel or alternative isoforms of the protein molecule. Differential splicing can now be studied genomewide using whole-transcript microarrays [2].

## 2. Variability in Gene Expression

The variability, thus created, in subcellular localization, in enzymatic activity, and particularly in degrees of stability of protein molecules, renders alternative splicing a source for diversity in cellular functionality and viability.

Disease entities arise in terms of such protein diversity engendered by extensive exon splicing, in further enhancing differential expression of individual genes. The combinatorial nature of regulation of alternative splicing is considerable and would call into operation both transregulatory splicing factors and cis-acting elements within the sequence structure of the pre-mRNA molecule. The organization of the exon-exon junction complex provides a link between biogenesis, nuclear export, and translation of the transcripts [3].

A basic concept of homeostatic equilibrium would characterize systems of evolution implicating such processes as phosphorylation of serine- and arginine-rich proteins that act as transfactors in alternative splicing.

The absence or presence of mutations that may be, at times, related to aberrant splicing patterns would indicate a mechanistic framework in further enhancing cellular diversity, linked closely to genomic regulation.

## 3. Expression Complexity

The complexity in gene expression appears to promote the important roles for splicing of pre-mRNA molecules in a manner that is illustrative of the potentially huge diversity at the level of RNA transcription. Transcriptional coupling to alternative splicing is a key feature of the DNA-damage response [4]. Translational and posttranslational modifications of protein structure are further mechanisms in the development of adaptive change in response to evolutionary need. Protein phosphorylation, in particular, appears implicated in the silencing of serine- and arginine-rich proteins as transfactors and might significantly promote exon exclusion from the pre-mRNA molecule sequence.

The general first principles in organization of exon sequence splicing enhancement and silencing appear of paramount importance in further elucidating the nature of mechanistic pathways that operate in cancerous transformation and malignancy. 

The transfactors in aberrant splicing of mRNA regulate in terms also of the evolution of several potential biomarkers in cancer. It would appear that the nature of protein diversity in neoplastic lesions arises largely in terms of aberrant splicing and often in the absence of correspondingly detectable mutations. Aberrant splicing may initiate abnormal exon skipping, intron retention or the introduction of new splice sites [5].

However, mutations may at times affect splicing sites and the spliceosome subcomponents in further development of engendered protein diversity [6].

Neoplastic transformation involving lesions such as carcinomas of the prostate and breast would indicate that protein diversity reflects the multimodal diversity in gene expression arising through the generation of mRNA isoforms, as these subsequently are transported between nucleus and cytosol. The combinatorial mechanics of protein:RNA binding would at times interfere with RNA:DNA binding in a manner further promoting exonic splicing enhancement or silencing.

The potentiality for involvement in diseases such as Duchenne muscular dystrophy and spinal muscular atrophy is also reflected in inherited disorders such as cystic fibrosis and beta-thalassemia. Antisense-mediated exon skipping is one of the most promising approaches for the treatment of Duchenne muscular dystrophy and has recently been shown to correct the reading frame and restore dystrophin expression in vitro and in vivo [7].

## 4. Combinatorial Regulation

The general principles of such combinatorial control in alternative splicing would arise as a derived parametric functionality of the constitutive splicing patterns in the first instance.

## 5. Aberrant Splicing

Aberrant splicing mechanics would secondarily alter the gene expression of several protein synthetic pathways in a manner predominantly correlative with the molecular stability and the intracellular localization of the synthesized protein molecules.

Referential indicators of the various roles of pathway generation would also arise within contexts of progressively active, aberrant splicing patterns.

Transposable elements are major sources of new exons in higher eukaryotes [8].

Exon skipping, in particular, helps illustrate the added roles of involvement by engendered nonsense mutations and stop condons in inducing absence of the protein or the production of a much truncated form of the molecule. Dystrophin, in particular, appears especially susceptible to the evolutionary modification in protein expression, as dimensionally expressed in terms either of Duchenne muscular dystrophy or else as Becker's muscular dystrophy.

Antisense oligosaccharides and the implied functionality or dysfunctionality induced by small nuclear ribonucleoprotein and by small interfering RNA molecules would appear examples of classic forms of influence that are potential targets in control of alternative or aberrant splicing.

## 6. Empiric Control of Splicing Events

Empiric control of splicing, with the use of administered small molecules, would indicate or earmark systems of variability in expression of individual genes.

Transcriptome comparison has been limited to differential analysis at the gene level instead of the individual transcript isoform level [9].

Contextual microenvironmental conditioning appears an essential operative series of systems in enhancing or silencing both constitutive and alternative splicing.

Multiple tau isoforms, as splicing alternatives in neurofibrillary tangle formation, would correlate with progression of dementia as seen with Alzheimer's disease and frontotemporal dementia, and also with various “tangle-only” dementias such as Pick's disease, corticobasal degeneration, progressive supranuclear palsy, and argyrophilic grain disease. Restricted tau isoform expression is related to a hyperphosphorylation of the tau molecule.

Microtubular assembly is impaired as a consequence of neurofibrillary tangle formation in terms relative to the emergence of other identifiable molecular species. Partly affected would appear to be the intraneuronal trafficking systems as readily indicated by axonal pathology.

## 7. Aggressive Neoplasia

The emergence of aggressive forms of neoplasia is exemplified particularly by the androgen-independent forms of prostatic adenocarcinoma that are progressively dedifferentiated as a consequence of possible aberrant splicing of pre-mRNA molecules. In terms of the added emergence of variably expressed protein molecules, the neoplastic lesion indicates the malignant transformation step in further modification of gene expression via aberrant splicing.

Spinal muscular atrophy is remarkable in the human as a consequence of the duplication of the survival of motor neuron gene involvement in terms of the SMN2 with exon 7 exclusion.

With reference to the modification of alternative splicing results it would appear that transfactors operate as contextual conditioning parameters within a setting especially of constitutive splicing. Indicators of the overall dimensions of the splicing mechanisms affecting the pre-mRNA would indicate protein diversity as a multifunctional motif in further enhancement or suppression of pathological endpathways [10].

The balance of factors controlling apoptosis may implicate Bcl isoforms, and their generation would characterize in strict terms the evolutionary dynamics of such lesions as neoplastic transformation and progression.

## 8. Malignant Neoplastic Change

Neoplastic malignant transformation appears to arise as a phenomenon secondarily characterizing attributes of alternative, aberrant splicing. The lack of mutations in such cases may presumably call into operation a serial modification of systems reflected as progression parameters, in tumor dedifferentiation and spread.

Progression of neoplastic transformation appears linked to a sequential promotion of various pathway effects as illustrated by a tendency for tumor dedifferentiation, concurrent with progression of spread of the neoplastic lesion. Methods to analyze gene expression and identify regulatory elements in BRCA 1/2 are now required to complement standard approaches to mutational analysis [11].

Suppressor genes and oncogenes, in general, would reflect the introduction of a series of modifying steps that augment protein diversity as indicated by diagnostic biomarkers of malignancy.

Spread of tumors is an incorporated system of mechanistic pathways that alternatively modifies gene expression, as parallel steps to evolving neoplastic progression.

## 9. Increasing Protein Diversity

Increasing splicing and increasing protein diversity, and protein molecular truncation, would further evolve as systems of recognizable identity within contextual change, as primary malignant transformation. An increasing proportion of mutations is now believed to influence m RNA splicing [12].

Indeed, the terms of reference of alternative splicing would arise within contexts of operatively constitutional change and also as possibly aberrant gene expression. Many alleles of human disease genes have mutations within splicing consensus sequences that activate cryptic splice sites [13]. Splicing further indicates the resolving dimensions of neoplastic progression as an expression of the splicing of diverse protein molecular species.

An example is the altered balance between Bcl-xL and Bcl-xS, two splice variants of the apoptosis regulator Bcl-x [14].

Combinatorial influence as systems of constitutive splicing would arise as predominant exclusion of introns and relative preservation of exons in splicing. 

The engine in manufacturing modified protein diversity indicates the constant operability of systems of change in progression of a given malignant neoplasm, both as a genetic lesion and also as a system of evolutionary character and transformability. Certain p53 isoforms are aberrantly expressed in various tumors strongly implying their involvement in tumorigenic events [15].

The given dimensional series of events reflects the real nature of the alternative, aberrant splicing mechanics, beyond actual biochemical identity of the skipped or included exon in question.

Diversity of acquired change in gene expression would hence constitute a realization of events as active components of the mechanistic splicing pathways.

Primary events in splicing are the true biochemical expression of a transforming identity in cancerogenesis and progression, both locally within the primary organ, and also systemically within the body. Altered RNA binding proteins appear to act as global generator of cancer-related RNA alternative splicing [16].

A multievolutionary set of principles seems to govern tumor progression in terms also of such systems as CD44 that acts prominently as cell adhesion pathways [17].

Stromal infiltration is, hence, the overall characterization of a series of potential splicing events that contextually incorporate alternative pathways, with aberrant systems of progression as increasing protein diversity in molecular synthesis.

Overall dimensions are the product of an involvement of events that modifies in sequential fashion the protein molecular characterization arising as primary splicing events.

Operative intracellular trafficking and the formation of intranuclear inclusions, and also the transport across the nuclear membrane, are systems of operative intervention and also secondary pathways leading to potentially diverse splicing events affecting messenger RNA.

The controlled expression of diverse isoforms of protein, in turn, regulates or modifies the expression of genes. Protein:RNA interaction and the modifications in RNA:DNA binding would indicate the potentiality for progression of lesions such as malignant neoplasms that adaptively induce modifications in protein constitutive identity and in cellular-matrix interactions such as those due to the local infiltration of tissues.

## 10. Concluding Remarks

The lack of recognizable mutation in many instances of alternative splicing creates an environment for diversity that is reflected secondarily, and also quantitatively, in molecular modification.

The physicochemical attributes in lesion creation indicate a contextual diversity that is modifiable in terms of neoplastic progression.

Hence, the primary nature of evolving neoplastic lesions is further classifiable in terms of the attributes of the primary or predominant splicing events.

Induced referential systems of such neoplastic change are illustrative of the new attributes that sequentially promote further aberrant splicing events, as dictated by evolutionary progression of the lesion both locally and systemically.

Dimensions of involvement by splicing of exons, with predilected definition of the exon-intron junctions, the ligation of the individual exons, and the inclusion or exclusion of exons, require a dynamic interactivity of cis-acting factors and transregulatory factors.

The involvement implicates interactions of gene transcriptional activity within a context of production of the pre-mRNA as directly conducive to the specific exonic sequence of the resultant messenger RNA transcript.

The inherent susceptibility of the exon/intron sequences to splicing is implicated as a poorly understood parameter in the overall phenomenon of splicing of premessenger RNA.

It would appear that turnover dynamics allow for the permissive operation of both cis- and transfactors that coherently incorporate constitutive and regulated splicing in a manner that actually promotes alternative regulatory splicing. In this sense, the spliceosome engine is a regulatory mechanism in its own right that promotes further control at multiple operative levels in the executive dynamics of splicing interventions.

In terms, therefore, of the systematic manipulative interventions of splicing, there might be introduced assembly of the spliceosome that, in a specific manner, directly promotes deterministic action of several transacting factors, as seen in neoplastic evolution.

Clear-cut indicators of such transacting factors would operate as a modulating series of pathways primarily implicating cis-factors also. Inherent susceptibility of exonic and intronic elements, to activation and, particularly, to inhibition, would operate as contextual sequences of enhancement or silencing of the splicing events.

A primacy in the operability of the exonic and intronic sequence silencing mechanisms would hence be contextually relevant in terms of a sequence-manipulating series of conditioning mechanics.

It is with reference to cis-factors, therefore, that constitutive splicing would carry significant power in the execution of forms of splicing directly conducive to the production of a final messenger RNA transcript that translocates from the nucleus to the cytoplasm. In this connection, the phosphorylation of protein components of the spliceosome, such as the SR proteins, would indicate a further operative involvement of other components of the spliceosome, particularly the heterogeneous nuclear RNA, as transnuclear membrane translocation.

Protein phosphorylation is, hence, both a generally applicable modifying step in protein interactivity or reactivity and also specific modulatory mechanisms in the localization dynamics of the protein-RNA complex within the cell.

It is in terms of ongoing contextual setting of the exonic and intronic silencing mechanics of splicing that the main modulatory systems in splicing would contribute by transfactors in tumor evolution and spread.

The marked diversity of protein molecular phenotype is significant in terms of such contextual sequences that promote overall dynamics of turnover of protein molecules. Both direct and also indirect posttranslational modifications of protein molecules would implicate subsequent consequences in molecular stability, enzymatic activity, and also localization specifics within the cell.
